# BZW2/5MP1 acts as a promising target in hepatocellular carcinoma

**DOI:** 10.7150/jca.53282

**Published:** 2021-06-22

**Authors:** Guoxiong Li, Anqian Lu, Anna Chen, Shuang Geng, Yu Xu, Xin Chen, Jin Yang

**Affiliations:** 1 Department of Gastroenterology, Affiliated Hospital of Hangzhou Normal University, Hangzhou Normal University, Hangzhou, Zhejiang 310015, P.R. China.; 2 Department of Translational Medicine Center, Affiliated Hospital of Hangzhou Normal University, Institute of Hepatology and Metabolic Diseases of Hangzhou Normal University, Hangzhou, Zhejiang 310015, P.R. China.

**Keywords:** Hepatocellular carcinoma, BZW2, Prognosis, Translation

## Abstract

Basic leucine zipper and W2 domain 2 (BZW2), also known as 5MP1, is a protein related to translation regulation. Evidence from previous studies indicates that BZW2 is involved in tumorigenesis in several cancers. However, little is known about the role of BZW2 in hepatocellular carcinoma (HCC). In this study, we first analyzed the gene expression profile of BZW2 in multiple HCC datasets. Next, we explored the biological effects of BZW2 in HCC cell lines. BZW2 was overexpressed in different HCC cohorts. Multivariate analysis confirmed that increased BZW2 expression is an independent prognostic indicator of shorter overall survival. BZW2 coexpressed genes were mainly enriched in the biological processes of ribonucleoprotein complex biogenesis, rRNA metabolism, translational initiation, and negative regulation of metabolic processes. BZW2 depletion reduced proliferation, clonality, and invasion and increased apoptosis in MHCC97-H cells. Furthermore, BZW2 overexpression or knockdown enhanced or impaired c-Myc expression, respectively. Overall, these findings identified BZW2 as a biomarker of HCC and provided novel insight that the effect of BZW2 on the translatome is a potential mechanism that promotes HCC progression via the c-Myc pathway.

## Introduction

Human hepatocellular carcinoma (HCC) is the third-leading cause of cancer-related death worldwide 1. Despite great advances in the management of HCC, the mortality rate remains high. There is still a great need to identify new biomarkers and develop novel treatment strategies for HCC.

Basic leucine zipper and W2 domain 1 (BZW1) and BZW2, also known as eIF5-mimic protein (5MP) 2 and 1, respectively, are human proteins previously characterized as transcription factors belonging to the basic-region leucine zipper (bZIP) superfamily 2. Accumulating evidence has gradually determined the role of BZW2 in tumor biology. For instance, BZW2 expression is higher in the tumor tissues of muscle-invasive bladder cancers than in normal tissues. Knocking down BZW2 inhibits cell cycle progression and proliferation and induces apoptosis 3. BZW1 and BZW2 were reported to promote the tumor growth of salivary mucoepidermoid carcinoma 4 and fibrosarcoma 5, respectively. More recently, it was found that BZW2 is highly expressed in liver tumor tissues 6 and colorectal cancer tissues 7. These findings suggest that aberrant BZW2 expression might be a specific marker for some cancers.

Despite their original discovery in 2001 as transcription factors, accumulating evidence indicates that BZW1 and BZW2 are translation regulatory proteins. Translation is a key regulatory step linking mRNA and proteins 8, 9. In various human cells, the non-AUG initiation rate is nearly constant under a fixed nucleotide context 10, 11, and this range of non-AUG initiation is controlled in part by BZW2. BZW2 is a competitor or mimic of eukaryotic translation initiation factor 5 (eIF5), which prepares for initiation through its GTPase activation protein (GAP) function and yet prevents eIF1 from anchoring to the initiating ribosome 12. Due to the role of the interaction of BZW2 with eIFs and its inhibition of general and gene-specific translation in multiple mammalian systems, the hypothesis was raised that the homeostasis of the non-AUG translatome is maintained through the coordinated expression of BZW2 and eIF5 10. In fact, the pro-oncogenic properties of BZW2 have been associated with the translation of the transcription factor ATF4 through the process whereby reinitiation is delayed based on upstream open reading frames 5, thereby enhancing the survival of cancer cells under stress conditions. More recently, BZW2 was proposed to reprogram c-Myc translation in favor of generating a more oncogenic AUG-initiated isoform rather than a less oncogenic CUG-initiated isoform 7.

Here, we systematically analyzed BZW2 expression in multiple HCC datasets and found that the abnormal expression of BZW2 was closely associated with HCC prognosis. The biological effect of BZW2 might occur via a translation regulatory mechanism by which BZW2 modulates eIF factors through c-Myc signaling.

## Materials and methods

### Data acquisition and processing

The Cancer Genome Atlas (TCGA) - Liver hepatocellular carcinoma (LIHC) cohort was obtained from cBio Cancer Genomics Portal (c-BioPortal) (http://cbioportal.org) 13 and analyzed in databases, such as GEPIA (http://gepia.cancer-pku.cn/) 14. In GEPIA, analysis of the relative expression of genes in tumor and adjacent tissues or normal tissues from the Genotype-Tissue Expression database (GTEx) (https://gtexportal.org/) 15 was performed. Raw GSE14520, and GSE76427 data were obtained from the Gene Expression Omnibus (http://www.ncbi.nlm.nih.gov/geo/) and processed as previously described 16, 17. In addition, HCCDB, a database of HCC expression atlases including curated public HCC expression datasets that include approximately 4000 clinical samples, was used to explore HCC gene expression 18. Tumor purity was assessed by the TIMER database 19.

### Coexpression analysis

BZW2 coexpression analysis was performed by the LinkedOmics database (http://www.linkedomics.org/) 20. GO biological process (GO), Kyoto Encyclopedia of Genes and Genomes (KEGG) pathways, kinase-target and transcription factor-target enrichment were performed by gene set enrichment analysis (GSEA) 21.

### Human HCC samples and immunohistochemical analysis

A total of 11 patients with HCC who underwent surgical resection of a primary tumor at the Affiliated Hospital of Hangzhou Normal University were enrolled in this study. Formalin-fixed paraffin-embedded sections were obtained from the Affiliated Hospital of Hangzhou Normal University Department of Pathology. All the protocols used in this study were approved by the local ethics review board of the affiliated hospital of Hangzhou Normal University (2019(02)-HS-52).

For immunohistochemical studies, the primary antibody was a rabbit polyclonal BZW2 antibody (1:50 dilution; ab254772, Abcam). The sections were evaluated at 100× and 400× magnifications to identify areas where even BZW2 staining was observed.

### Cell culture and transfection

The HCC cell line MHCC97-H was cultured in DMEM supplemented with 10% fetal bovine serum (FBS), 100 U/mL penicillin, and 0.1 mg/mL streptomycin. All the cells were maintained in a humidified atmosphere containing 5% CO_2_ at 37 °C.

Short hairpin RNA (shRNA) targeting human BZW2 (sh-BZW2) and nonspecific control shRNA (sh-Ctrl) were synthesized by TsingKe Inc., China. The sequence of the shRNA targeting BZW2 was TGATGTTCTGAGCGAAGAA. An overexpression plasmid was constructed by inserting the open reading frame of BZW2 into the pcDNA3.1 vector between the HindIII and BamHI sites. These vectors were separately transfected into cells with Lipofectamine 2000 (Invitrogen, Carlsbad, CA) according to the manufacturer's instructions.

### RNA extraction and qRT-PCR

Total RNA was purified using TRIzol (Invitrogen, Carlsbad, CA). cDNA was synthesized using the PrimeScript™ RT reagent Kit (Takara, Beijing, China). qRT-PCR was performed using SYBR Master Mixture (Takara, Beijing, China) on an ABI7900 PCR system. The primer sequences were as follows: BZW2 forward, CATTGCGGCCTCATTTGCTG and reverse, CGGAGGAAGTCGGAAAGCTC; c-Myc forward, CATCAGCACAACTACGCAGC and reverse, GCTGGTGCATTTTCGGTTGT; and GAPDH forward, AGCCACATCGCTCAGACAC and reverse, GCCCAATACGACCAAATCC.

### Protein extraction and Western blot analysis

Total protein was extracted using radioimmunoprecipitation assay (RIPA) buffer containing protease and phosphatase inhibitors (Beyotime, China). The protein samples were separated using SDS-PAGE and transferred to PVDF membranes, and the membranes were blocked with 5% skim milk. The membranes were probed with primary antibodies at 4 °C, followed by incubation with HRP-conjugated secondary antibodies before visualization by ECL kits (Thermo Scientific, USA). In total, the primary antibodies included those specific for BZW2 (ab254772, Abcam), c-Myc (ab32072, Abcam), and GAPDH (10494-1-AP, Proteintech). GAPDH was used as the loading control.

### Cell proliferation and colony formation assays

Cell proliferation was assessed by CCK-8 assay (Dojindo Laboratories, Japan). Cells were seeded into 96-well plates at a density of 2×10^3^ cells per well. CCK-8 reagent was added to each well at the indicated time points. The absorbance at 450 nm was detected with a microplate reader (BioTek, USA). For the colony formation assay, after culturing for 2 weeks, the colonies on the plates were fixed in 4% paraformaldehyde and stained with 1% crystal violet, and the numbers of colonies were counted. The experiments were conducted in triplicate.

### Cell cycle and apoptosis analysis

For cell cycle analysis, the cells were trypsinized and fixed with 75% ethanol. The cells were washed and resuspended in 300 µl DNA staining solution (Multiscience, China), followed by analysis using a FACSCalibur flow cytometer. Cell apoptosis was detected by using the Annexin V-FITC/Propidium Iodide (PI) Apoptosis Detection Kit (BD Biosciences, USA) according to the manufacturer's instructions. Each measurement was performed in triplicate.

### Wound healing and invasion analysis

Cells were cultured in 6-well plates at a seeding density of 5×10^4^ cells/well. When cells reached approximately 100% confluence, a straight wound was made using a sterile pipette tip. Phase-contrast images were captured. For the invasion assays, serum-free medium was added to the upper Transwell chambers (8-μm pore size; Corning, NY, USA), and medium with 5% FBS was added to the lower chamber as a chemoattractant. All the experiments were performed in triplicate.

### Statistical Analysis

The Kaplan-Meier method with a two-sided log-rank test was used to compare the overall survival (OS) time or disease-free survival (RFS) time of the patients in the high- and low-risk groups, and these groups were separated according to the median cutoff of gene expression. The Cox proportional hazards regression model was used to calculate the hazard ratios (HRs) and their 95% confidence intervals (CIs). All the statistical analyses were conducted using R v3.5.1 (https://www.r-project.org/) or GraphPad Prism (GraphPad Software Inc., San Diego, CA).

## Results

### BZW2 is widely upregulated in HCC tissues

**Figure 1:**
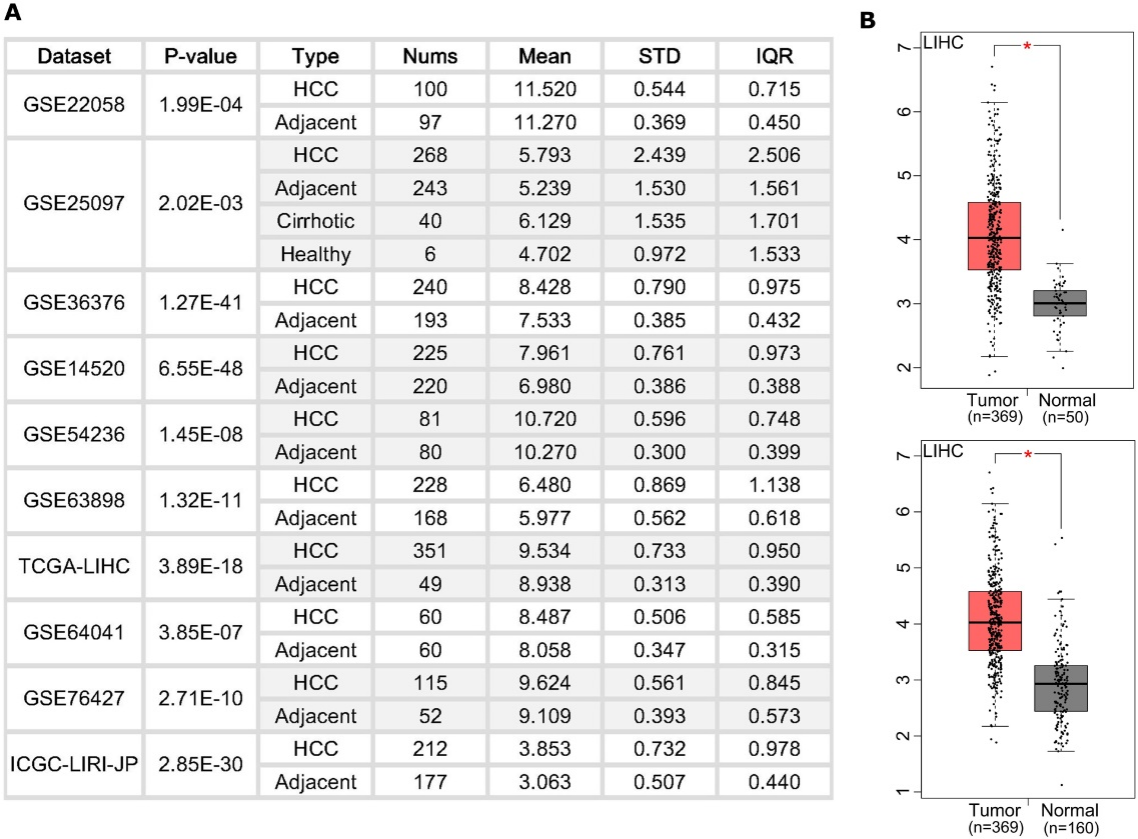
** The expression of BZW2 in tumor tissues and adjacent tissues.** (**A**) Chart showing the expression of BZW2 in different cohorts. (**B**) In the LIHC cohort, BZW2 in tumor tissues was compared with adjacent tissues (upper panel) or with the matched LIHC normal and GTEx data (lower panel).

We first examined the BZW2 expression level in HCC patients (**Figure 1A**) from the HCCDB database 18. Ten cohorts showed a higher expression level of BZW2 in HCC tumors. Specifically, we checked the expression profile of BZW2 in the LIHC cohort using normal liver data from GTEx as the control (**Figure 1B**). All the results indicated that BZW2 is significantly more upregulated in tumor tissues than in adjacent tissues.

### BZW2 expression is related to clinicopathology

**Figure 2:**
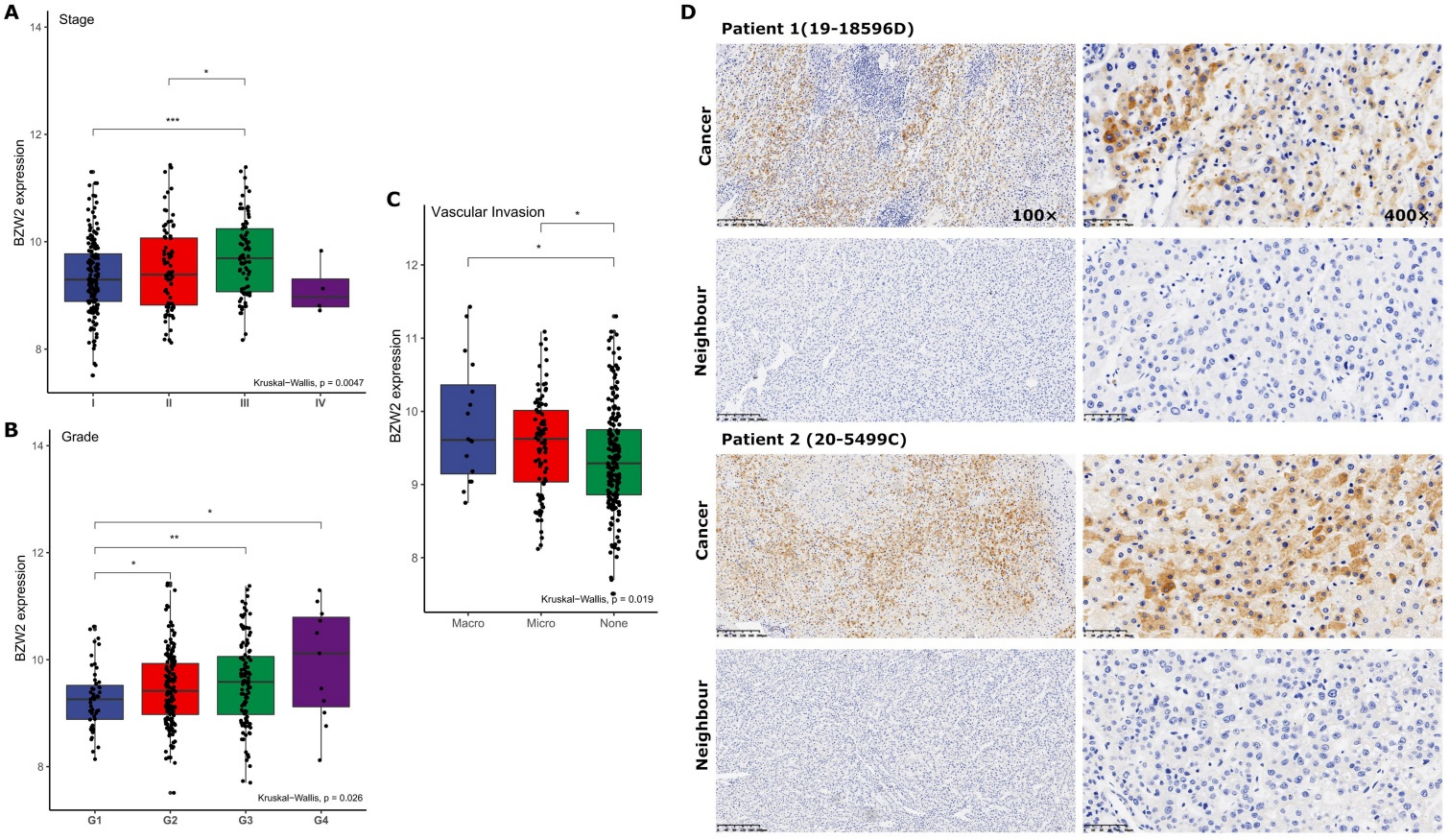
** BZW2 expression varied in patients with different clinicopathological characteristics.** (**A**) BZW2 expression in LIHC patients with different tumor stages. (**B**) BZW2 expression in LIHC patients with different histological grades. (**C**) BZW2 expression in LIHC patients with different vascular invasion levels. (**D**) Representative IHC images of BZW2 expression in cancer tissues and paracancerous tissues. *P < 0.05, **P < 0.01, ***P < 0.001.

We then compared the expression profile of BZW2 in HCC patients from the LIHC cohort with different clinicopathological characteristics. The results showed that BZW2 expression was significantly upregulated in advanced pathological stages (III) compared to the early stages (I/II) (**Figure 2A**) and was also higher in high-grade tumors and tumors with macrovascular invasion (**Figure 2B, C**).

Next, we determined BZW2 expression in 11 pairs of HCC tissues by immunohistochemistry (IHC) (**Figure 2D**). In accordance with the TCGA-LIHC data, IHC confirmed the upregulated protein level of BZW2 in HCC tissues and the cytoplasmic localization of BZW2, and the patients with high expression levels of BZW2 demonstrated a trend of having a more advanced disease state (**Table 1**).

### Elevated BZW2 expression is associated with poor survival

**Figure 3:**
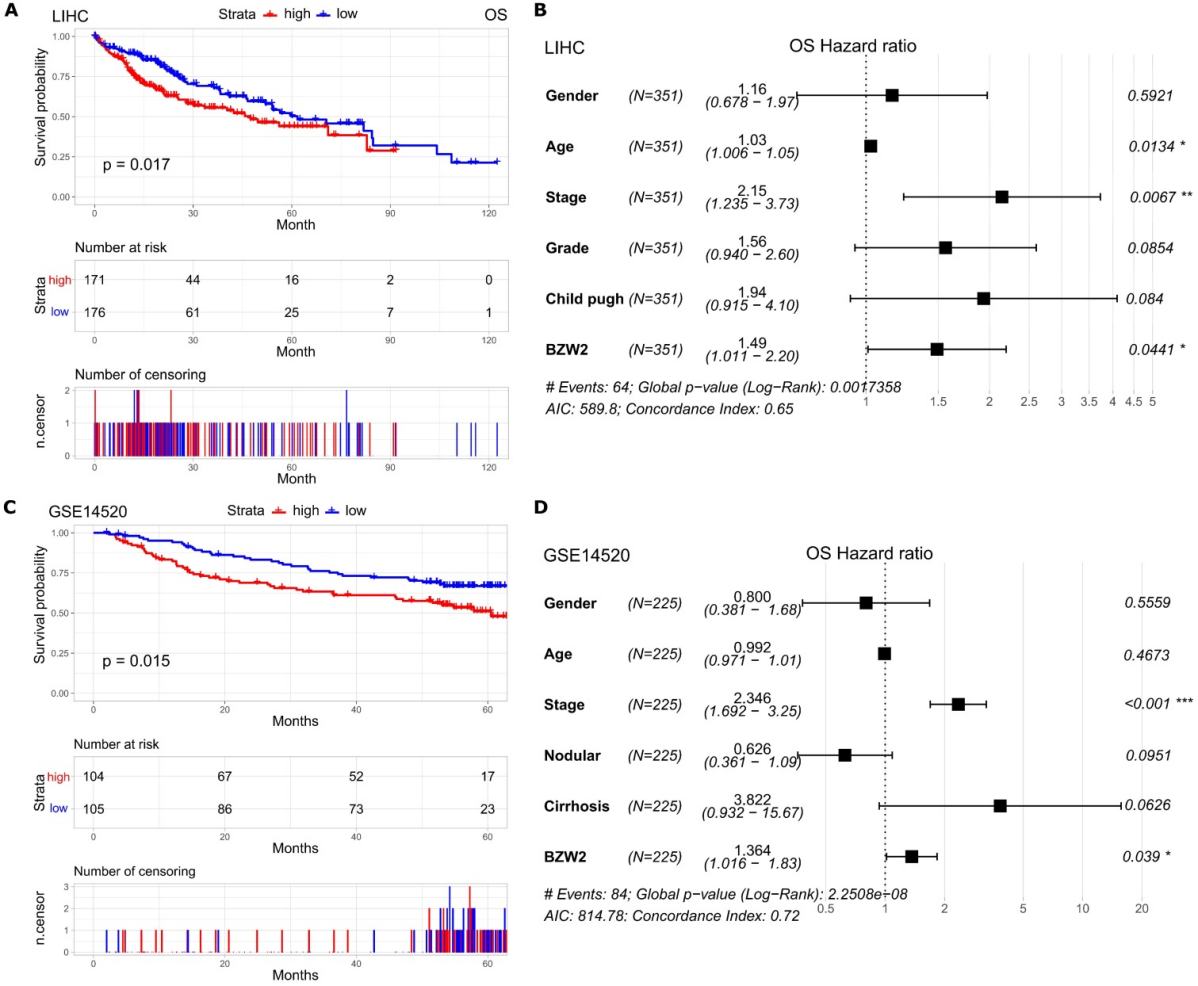
** Elevated BZW2 expression is associated with unfavorable outcomes in HCC.** (**A, C**) Kaplan-Meier curves of OS in the LIHC cohort and GSE14520 cohort, respectively. (**B, D**) Multivariable hazards models of BZW2 expression in the LIHC cohort and GSE14520 cohort, respectively.

Furthermore, we assessed the association between BZW2 expression and the survival outcomes of HCC patients. The high BZW2 expression group had a significantly shorter OS than the low expression group in the LIHC cohort (**Figure 3A**). In addition, high BZW2 expression resulting in poor survival was also verified in the GSE14520 cohort (**Figure 3C**).

To explore the independent prognostic value of BZW2 expression, multivariable hazards models were used to evaluate the impacts of BZW2 expression in the presence of varying clinical traits. BZW2 was associated with 1.49- and 1.36-fold higher risks of OS in the LIHC and GSE14520 cohorts, respectively (**Figure 3B, D**).

### Coexpression mode of BZW2

**Figure 4:**
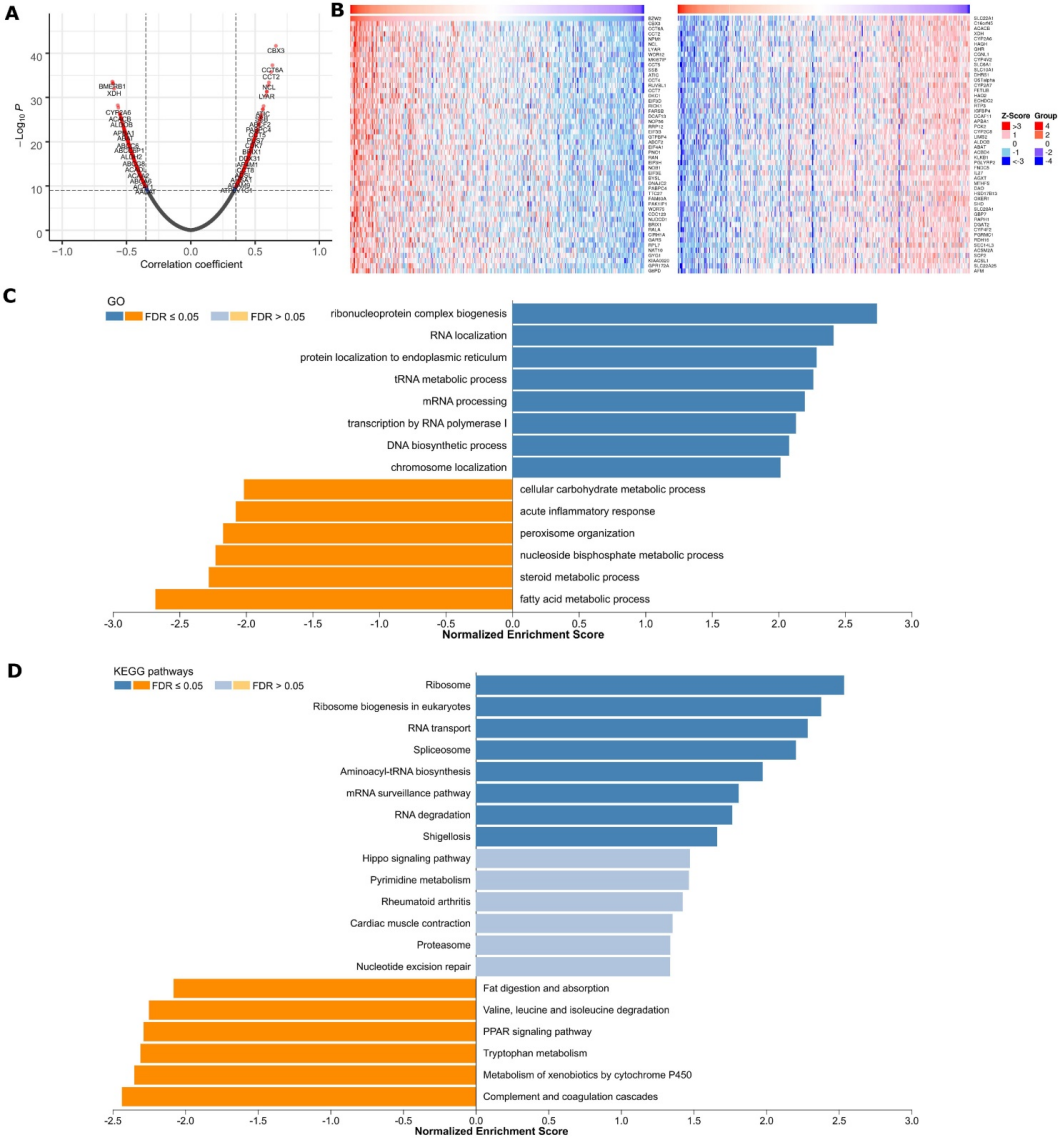
** BZW2 coexpression pattern in the LIHC cohort (LinkedOmics).** (**A**) Volcano plot of BZW2-highly correlated genes. (**B**) Heatmaps showing the top 50 genes that were positively or negatively correlated with BZW2. (**C, D**) Significantly enriched GO terms and KEGG pathways of BZW2 coexpressed genes, respectively.

Genome-wide BZW2 coexpression mode was examined in the LIHC cohort. In total, 891 mRNAs were identified to be significantly associated with BZW2 (*P* < 1E-10) (**Figure 4A** and **Table S1**). The top 50 significant genes positively or negatively correlated with BZW2 are shown in the heat map (**Figure 4B**).

Significant GO term annotation by GSEA showed that BZW2 coexpressed genes participate primarily in ribonucleoprotein complex biogenesis, rRNA metabolic process, ncRNA processing, and translational initiation, while activities such as fatty acid metabolic process, small molecule catabolic process, and protein activation cascade were inhibited (**Figure 4C** and **Table S1**). KEGG pathway analysis showed enrichment in the Ribosome, Ribosome biogenesis in eukaryotes, RNA transport, Spliceosome, Aminoacyl-tRNA biosynthesis and cell cycle pathways (**Figure 4D** and **Table S1**).

Furthermore, to explore the regulators of BZW2 in HCC, we analyzed the kinase and transcription factor enrichment among BZW2 coexpressed genes. The top 5 most significant kinases were primarily related to CHEK1, AURKB, CDK2, ATR, and PLK1 (**Table 2**). In fact, all of these kinases were significantly highly expressed in tumor tissues and associated with the OS of HCC (**Figure S1**).

The GSEA enrichment of transcription factors was mainly related to the ETS transcription factor (ELK1), Yin Yang 1 (YY1), MYC (c-Myc), and E2F transcription factor families (**Table 2**). Indeed, ELK1 is involved in epithelial-mesenchymal transition in HCC, which is crucial for cancer cells to acquire chemoresistance 22. YY1 facilitates HCC cell lipid metabolism and tumor progression by inhibiting PGC-1β-induced fatty acid oxidation 23. One recent study identified an E2F-driven transcriptional program that was associated with the development and progression of HCC 24.

### BZW2 promotes the proliferation of HCC cells

**Figure 5:**
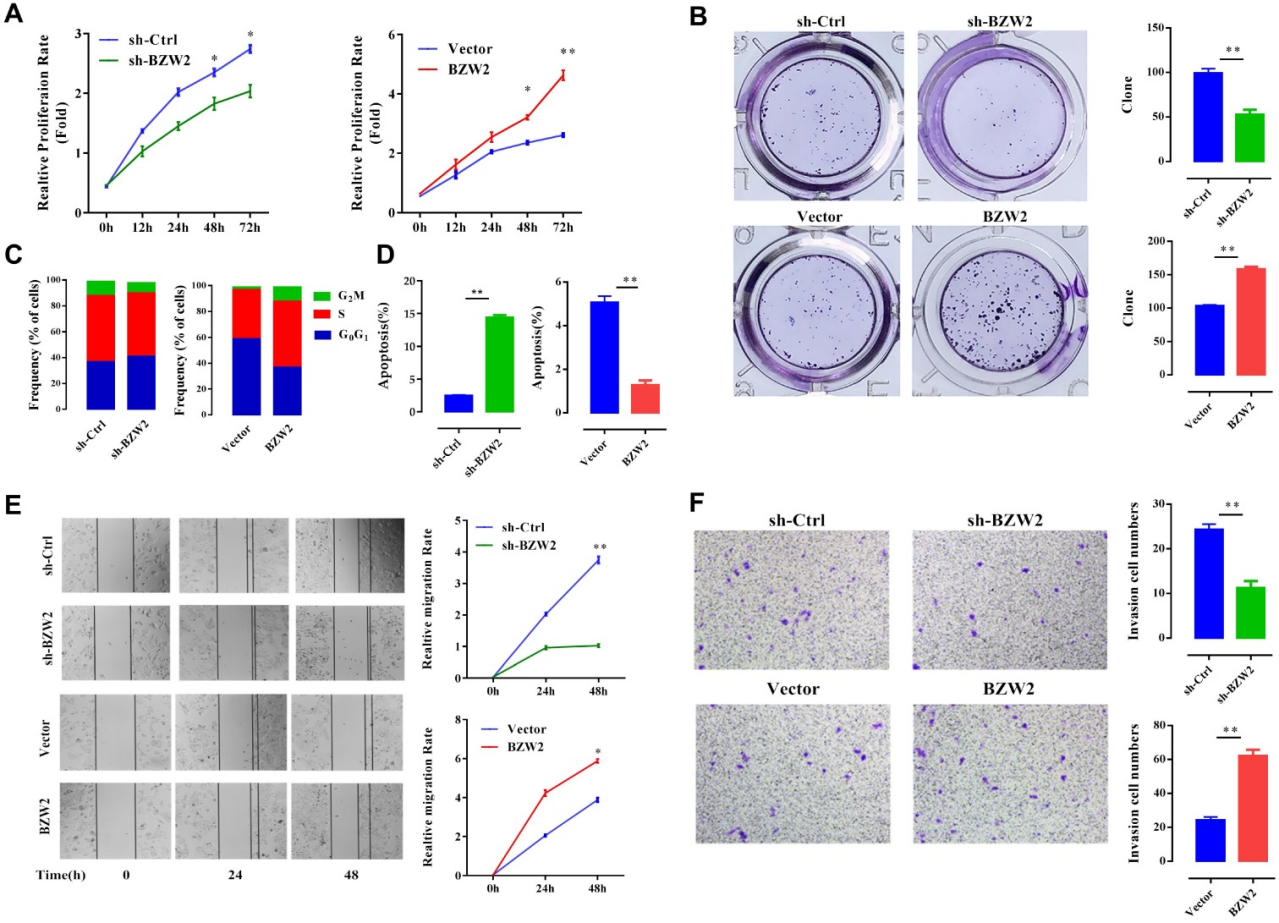
** BZW2 promotes the proliferation of HCC cells.** (**A**) The relative proliferation rate of transfected cells was determined by CCK-8 assay at the indicated times. (**B**) Photos of colony formation (left) and bar graph (right). (**C, D**) Effect of BZW2 depletion or overexpression on cell cycle distribution and cell apoptosis, respectively. (**E**) Photos (left) and bar plot (right) of migration in BZW2-overexpressing or BZW2-knockdown cells. (**F**) The invasion assay results after incubation in Transwell chambers for 48 h. The data are from three independent experiments and expressed as the mean ± SD. *P < 0.05, **P < 0.01.

We then examined the effect of BZW2 on the growth of HCC cells. The interference of BZW2 was validated by qRT-PCR, with a knockdown efficiency approaching 77.6%. CCK-8 assays indicated that BZW2 overexpression or knockdown significantly promoted or inhibited cell proliferation in MHCC97-H cells, respectively (**Figure 5A**). Furthermore, colony formation assays showed that BZW2 overexpression or knockdown substantially enhanced or impaired the colony-formation ability of the cells, respectively (**Figure 5B**).

We observed that the BZW2-transfected group had a higher proportion of cells in the S-phase than the control group. In contrast, BZW2 knockdown in MHCC97-H cells led to an increase in the G1 cell population, with a concomitant decrease in proportion of cells in the S-phase, indicating that BZW2 accelerated G1/S progression (**Figure 5C**).

Next, we quantified cell apoptosis and found that the number of apoptotic cells in the sh-BZW2 group was higher than that in the sh-Ctrl group. In contrast, ectopic BZW2 expression significantly inhibited the apoptosis of cells (**Figure 5D**).

In addition, we also observed a significant decrease in the migration or invasion of MHCC97-H cells after BZW2 gene silencing. Correspondingly, BZW2 overexpression restored the wound healing and invasion ability of HCC cells (**Figure 5E, F**). Collectively, these results suggest the oncogenic property of BZW2.

### BZW2-eIF5 interact in HCC

Since the non-AUG translation initiation rate is consistent across various organisms and oppositely regulated by eIF5 and BZW2 10, we next compared the expression of eIF5 and BZW2 in HCC.

**Figure 6:**
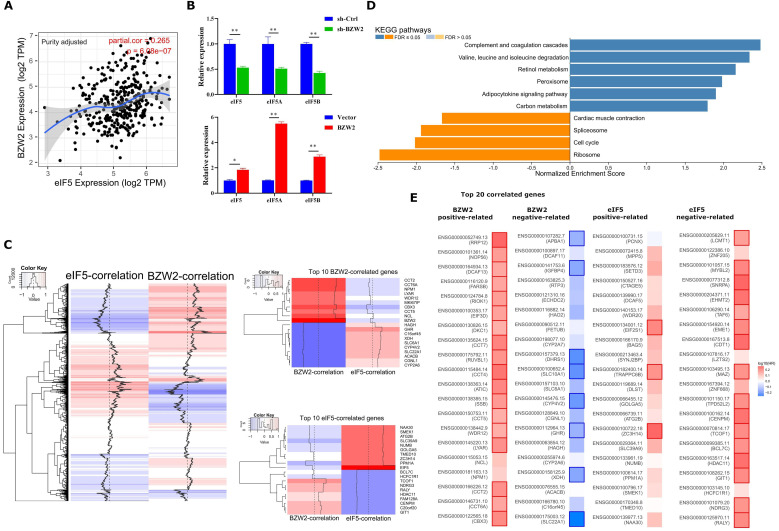
** BZW2-eIF5 interact in HCC.** (**A**) Correlation between BZW2 and eIF5 after tumor purity adjustment. (**B**) Expression changes in eIF5, eIF5A and eIF5B after BZW2 knockdown or ectopic expression. (**C**) Global heatmap of correlation coefficients of eIF5- and BZW2-associated genes. The right panel shows the enlarged heatmap of the top 10 BZW2- or eIF5-correlated genes. (**D**) GSEA plot of the KEGG pathways enriched in eIF5 coexpressed genes. (**E**) Survival map of the top 20 BZW2- or eIF5-correlated genes. The bold box denotes *P* < 0.05.

We first observed a positive correlation between the expression of BZW2 and eIF5 using the TIMER database (**Figure 6A**) and verified the coexpression mode using BZW2 overexpression or knockdown analysis (**Figure 6B**). Indeed, BZW2 showed a close relationship to genes belonging to the eIF family, such as eIF5A, eIF5B, eIF3a, eIF3b, and eIF3e (**Figure S2**).

We then performed eIF5 global coexpression analysis using LinkedOmics 20 (**Table S2**). The genes regulated by BZW2 showed a genome-wide, inverse correlation with genes regulated by eIF5 in terms of mRNA expression (**Figure 6C**). Comparing the relationship of survival prognosis with the top 20 coexpressed genes in the BZW2 or eIF5 group, most genes positively related to BZW2 were associated with high risk, while genes negatively related to BZW2 were associated with low risk. In contrast, most genes negatively related to eIF5 were associated with high risk in HCC (**Figure 6E**).

When exploring the functional meaning of eIF5 coexpressed genes, the GSEA enrichment of KEGG pathways was positively related mainly to the complement and coagulation cascades, valine, leucine and isoleucine degradation, and fatty acid degradation and negatively related mainly to ribosomes, DNA replication, and the cell cycle (**Figure 6D** and**Table S2**); these results are opposite the results of BZW2 (**Figure 4C**).

### c-Myc is the effector of BZW2-mediated HCC progression

**Figure 7:**
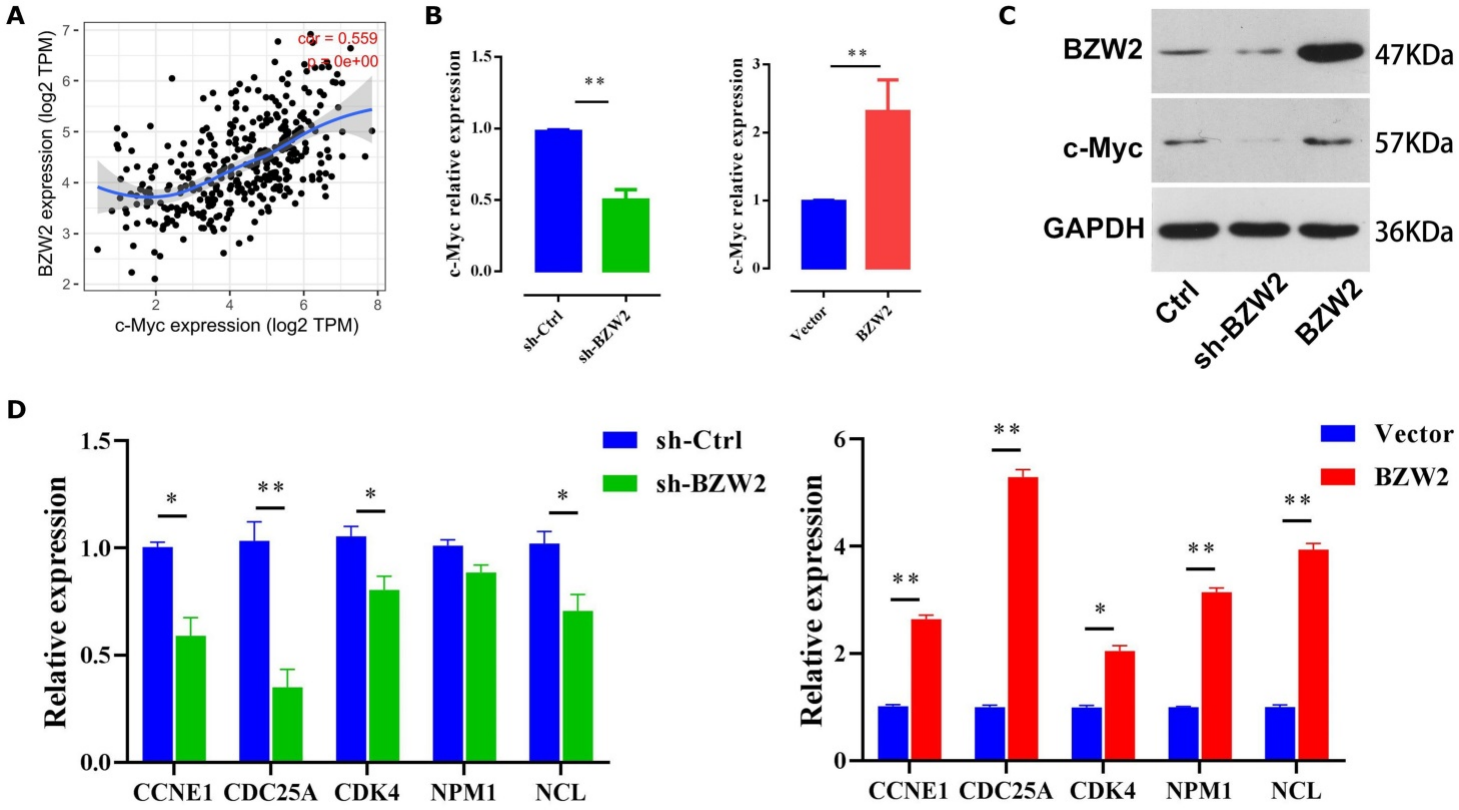
** BZW2 mediates c-Myc dysregulation.** (**A**) Correlation between BZW2 and c-Myc after tumor purity adjustment. (**B**) Expression changes in c-Myc after BZW2 knockdown or ectopic expression. (**C**) Western blot of BZW2 in the cells transfected with the indicated vectors. (**D**) Expression changes in c-Myc target genes after BZW2 silencing or overexpression.

As c-Myc is an important oncoprotein, we next investigated whether c-Myc participates in BZW2-mediated HCC cell proliferation. The expression of BZW2 showed a high correlation with that of c-Myc (**Figure 7A**). Furthermore, the GSEA results (**Table 2**) prompted us to hypothesize that BZW2 might regulate c-Myc expression.

Specifically, BZW2 depletion significantly reduced the expression of c-Myc in HCC cells at both the mRNA and protein levels. Following the increase in BZW2 expression, c-Myc expression exhibited an increasing trend in MHCC97-H cells (**Figure 7B, C**).

c-Myc functions as an essential regulator of G1/S transition by promoting the genes that drive cell cycle progression and cell growth 25. Consistent with these data, BZW2 depletion reduced several c-Myc target genes that are involved in cell cycle progression, e.g., CCNE1, CDC25A, and CDK4 (**Figure 7D**). Furthermore, qRT-PCR analysis demonstrated that several c-Myc target genes, such as NPM1 and NCL, were significantly upregulated after BZW2 upregulation (**Figure 7D**). Indeed, NPM1 is weakly expressed in hepatocytes and highly induced in HCC. Enhanced NPM1 expression has a clear correlation with increased tumor grade and poor prognosis 26. As a eukaryotic nucleolar phosphoprotein, NCL is involved in the synthesis and maturation of ribosomes. Increased levels of NCL confer aggressive tumor progression and poor prognosis in HCC 27. Thus, these results suggest that BZW2 might promote HCC growth via c-Myc signaling.

## Discussion

It was recently shown that BZW2 is a novel oncogene involved in several types of cancers 3, 6, 7, 28. Here, this study provided evidence that BZW2 is widely upregulated in liver tumor tissues. Furthermore, a high expression level of BZW2 correlates with a poor prognosis in HCC. Multivariate analysis confirmed that increased BZW2 expression was an independent prognostic indicator of shorter survival. More recently, one study indicated that silencing BZW2 in HCC BEL-7404 and HepG2 cells prohibits cell progression, as determined by suppressed proliferation, clonality, and invasion and increased apoptosis 6. Consistent with these data, we found that BZW2 deregulation affects the oncogenic property of BZW2 in MHCC97-H cells. These findings suggest that BZW2 might serve as an oncogene and promising biomarker of HCC.

This study revealed that BZW2 was correlated with the biological processes and signaling pathways involved in HCC pathogenesis. First, by BZW2 coexpression analysis, we revealed that in HCC, the expression of BZW2 is significantly associated with biological processes, such as rRNA processing, translational initiation, and nuclear-transcribed mRNA catabolic process, as well as with KEGG pathways, such as Ribosome and RNA transport. Second, mRNAs that are highly correlated with BZW2 showed a tendency to affect survival outcome. For most genes that are positively correlated with BZW2 (**Figure 4A, B**), functional assays showed that CBX3 overexpression promotes HCC cell proliferation 29. High CCT6A expression has potential value in the prognosis colorectal cancer liver metastasis 30. In addition, we noted an overall cytoplasmic localization of BZW2, which as consistent with a similar finding in colorectal cancer 7 and with the idea that this protein functions as a translation regulatory protein in the cytoplasm 31.

Translational control through start codon selection is considered a crucial driving factor in cancer development 7. One strategy of this reprogramming is the regulation of translation-related proteins that mimic initiation factors 32. As a translational regulatory protein, BZW2 affects the accuracy of translation initiation by counteracting eIF5, and this competition between eIF5 and BZW2 determines the noncanonical initiation rate genome-wide 10. The results of this study demonstrated that BZW2 and eIF5 were coexpressed in HCC. Moreover, BZW2 and eIF5 have opposite impacts on global gene expression. The survival map indicated that the top BZW2- or eIF5-related genes showed opposite trends in survival prognosis. These findings help to explain the potential contribution of BZW2 upregulation to the malignant behaviors of HCC. In agreement with the redefinition of BZW/5MP as a translational regulator, the basic leucine zipper portion of the protein exhibits strong similarity to a type of α-helical HEAT domain, which makes the whole protein similar to the C-terminal third domain of eIF4G 31, 33. This fact and further characterization of this protein as an eIF5 mimetic led the Asano group to rename this protein an eIF5-mimic protein (5MP) 20.

c-Myc is a master transcription factor that regulates the expression of 10%-15% or more of all cellular genes in a wide variety of cancer types 34. It has been reported that the conditional inactivation of c-Myc can be sufficient to induce sustained regression of HCC 35. In fact, c-Myc affects the activity of translational control factors 36. It was reported that BZW2 acts as an oncogenic driver gene and controls the selection of translational start codons of the c-Myc oncogene in colorectal cancer 7. In this study, we confirmed that BZW2 and c-Myc expression was positively correlated and that BZW2 upregulation increased the expression of c-Myc, suggesting that BZW2 might modulate tumor cell growth in HCC by regulating the c-Myc pathway.

In summary, our study identified BZW2 as a novel marker for HCC and revealed a mechanism by which BZW2 acts as an oncogene in the progression of HCC by mediating the expression of c-Myc. These findings suggest that BZW2 could be a valuable prognostic biomarker and a potential therapeutic target in HCC.

## Supplementary Material

Supplementary figures and table legends.

Supplementary table 1.

Supplementary table 2.

## Figures and Tables

**Table 1:** Correlation of BZW2 expression with clinicopathological parameters of human HCC patients

Clinicopathological parameters	No. of cases	Average BZW2 density	*P* value
**Stage**			0.049
I	4	0.179 ± 0.016	
II	7	0.228 ± 0.013	
**Grade**			0.247
I-II	6	0.202 ± 0.021	
III	5	0.222 ± 0.010	
**Portal vein tumor thrombin**			0.398
No	7	0.202 ± 0.014	
Yes	4	0.227 ± 0.024	
**Tumor size (cm)**			0.553
<5	9	0.208 ± 0.015	
>5	2	0.225 ± 0.019	
**Serum AFP (ng/ml)**			0.168
≤100	6	0.195 ± 0.014	
>100	5	0.231 ± 0.019	

The data are presented as the mean ± standard deviation (SD). *P* values were determined with a two-tailed *t* test.

**Table 2:** Kinase- and transcription factor-target networks of BZW2 in HCC

Enriched Category	Geneset	Normalized Enrichment Score	FDR
Kinase Target	Kinase_CHEK1	2.026	0.00E+00
	Kinase_AURKB	1.950	1.11E-03
	Kinase_CDK2	1.872	1.55E-03
	Kinase_ATR	1.874	1.66E-03
	Kinase_PLK1	1.878	2.22E-03
Transcription Factor	V$YY1_Q6	1.944	0.00E+00
	SCGGAAGY_V$ELK1_02	1.932	0.00E+00
	GCCATNTTG_V$YY1_Q6	1.883	7.10E-04
	CACGTG_V$MYC_Q2	1.826	1.85E-03
	V$E2F_02	1.812	1.90E-03

## Abbreviations

BZW2: basic leucine zipper and W2 domain 2
HCC: hepatocellular carcinoma
bZIP: basic-region leucine zipper
GAP: GTPase activation protein
eIF5: eukaryotic translation initiation factor 5
TCGA: The Cancer Genome Atlas
LIHC: Liver hepatocellular carcinoma
GEO: The Gene Expression Omnibus
GO: Gene ontology
KEGG: Kyoto Encyclopedia of Genes and Genomes
GSEA: Gene-set enrichment analysis
shRNA: short hairpin RNA
PI: propidium iodide
RIPA: radioimmunoprecipitation assay
OS: overall survival
RFS: disease-free survival
HR: hazard ratio
CI: confidence interval
IHC: immunohistochemistry
qPCR: Quantitative real-time PCR
