# Supplementary material for: BZW2/5MP1 acts as a promising target in hepatocellular carcinoma
Source: J Cancer. 2021 Jun 22;12(17):5125–35. doi: 10.7150/jca.53282 (PMC8317536; doi:10.7150/jca.53282)
Supplement: Supplementary file 1 — Supplementary figures and table legends. [file jcav12p5125s1.pdf]

Supplementary Table S1. GO terms and KEGG pathway enrichment of BZW2 coexpressed genes.

Supplementary Table S2. KEGG pathway enrichment of eIF5 coexpressed genes.

Supplementary Figure S1. Expression of BZW2-related regulators and association with survival outcome. (**A**) Top 5 kinase regulators of BZW2 coexpressed genes. All of these kinase genes were significantly highly expressed in tumor tissues. All these genes have a significant association with OS in HCC. (**B**) Transcription factors of BZW2 coexpressed genes.

Supplementary Figure S2. The expression of BZW2 is highly correlated with that of eIF5, eIF5A, eIF5B, eIF3a, eIF3b, and eIF3e.

a

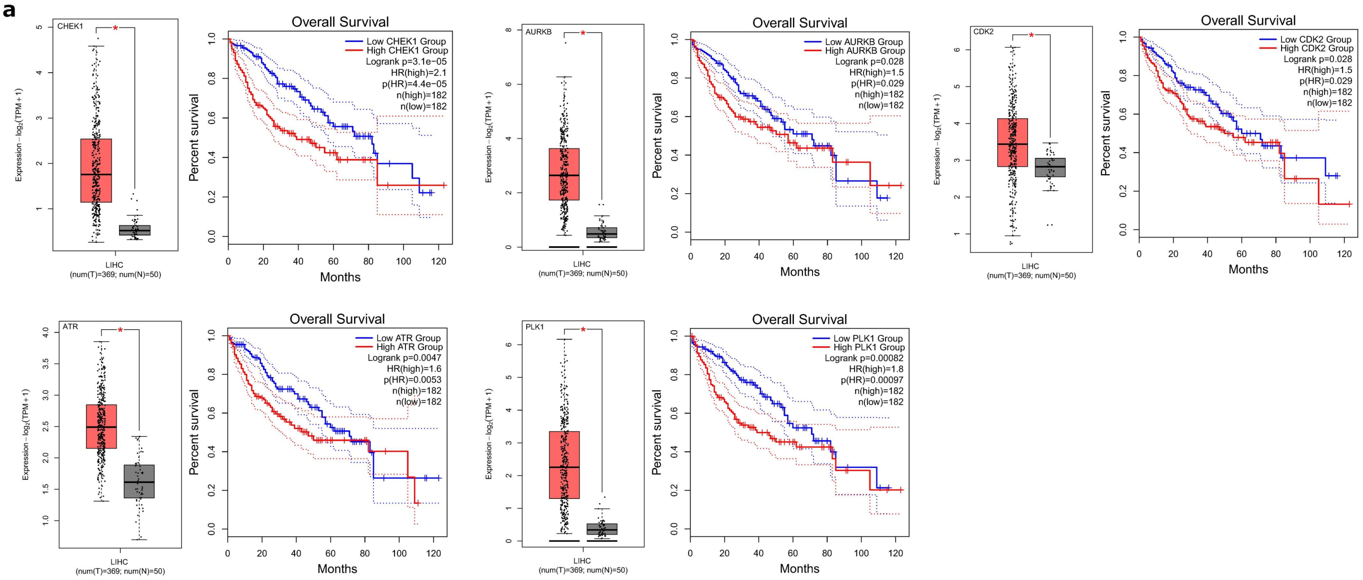
CHEK1
Expression - log2(TPM + 1)
LIHC
(num(T)=369; num(N)=50)
Overall Survival
Low CHEK1 Group
High CHEK1 Group
Logrank p=3.1e−05
HR(high)=2.1
p(HR)=4.4e−05
n(high)=182
n(low)=182
Percent survival
Months
AURKB
Overall Survival
Low AURKB Group
High AURKB Group
Logrank p=0.028
HR(high)=1.5
p(HR)=0.029
n(high)=182
n(low)=182
CDK2
Overall Survival
Low CDK2 Group
High CDK2 Group
Logrank p=0.028
HR(high)=1.5
p(HR)=0.029
n(high)=182
n(low)=182
ATR
Overall Survival
Low ATR Group
High ATR Group
Logrank p=0.0047
HR(high)=1.6
p(HR)=0.0053
n(high)=182
n(low)=182
PLK1
Overall Survival
Low PLK1 Group
High PLK1 Group
Logrank p=0.00082
HR(high)=1.8
p(HR)=0.00097
n(high)=182
n(low)=182

b

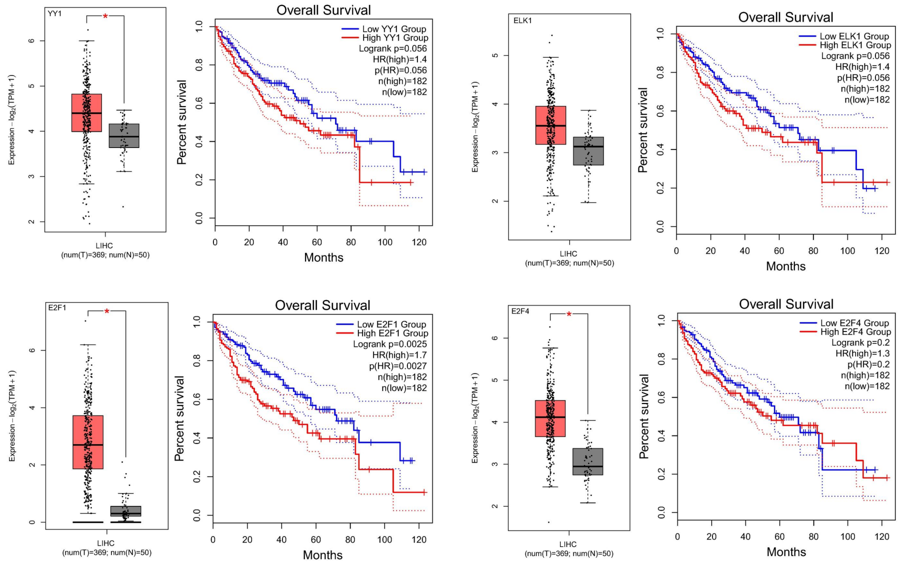
YY1
Expression - log2(TPM + 1)
LIHC
(num(T)=369; num(N)=50)
Overall Survival
Low YY1 Group
High YY1 Group
Logrank p=0.056
HR(high)=1.4
p(HR)=0.056
n(high)=182
n(low)=182
Percent survival
Months
ELK1
Overall Survival
Low ELK1 Group
High ELK1 Group
Logrank p=0.056
HR(high)=1.4
p(HR)=0.056
n(high)=182
n(low)=182
E2F1
Overall Survival
Low E2F1 Group
High E2F1 Group
Logrank p=0.0025
HR(high)=1.7
p(HR)=0.0027
n(high)=182
n(low)=182
E2F4
Overall Survival
Low E2F4 Group
High E2F4 Group
Logrank p=0.2
HR(high)=1.3
p(HR)=0.2
n(high)=182
n(low)=182

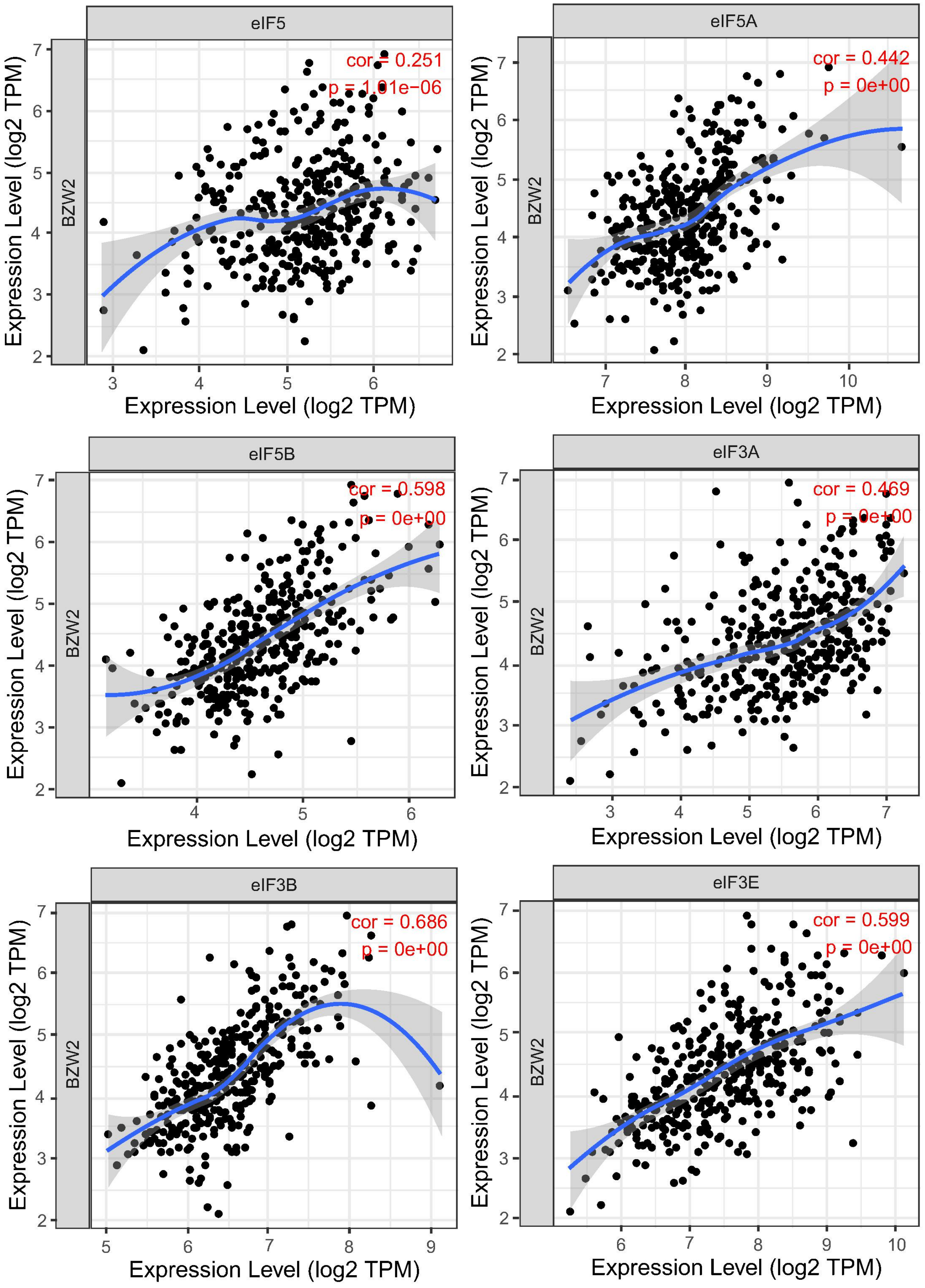
eIF5
cor = 0.251
p = 1.01e−06
eIF5A
cor = 0.442
p = 0e+00
eIF5B
cor = 0.598
p = 0e+00
eIF3A
cor = 0.469
p = 0e+00
eIF3B
cor = 0.686
p = 0e+00
eIF3E
cor = 0.599
p = 0e+00
BZW2
Expression Level (log2 TPM)
Expression Level (log2 TPM)
